# Multisensory integration across the menstrual cycle

**DOI:** 10.3389/fpsyg.2013.00666

**Published:** 2013-09-24

**Authors:** Sebastian Ocklenburg, Claudia C. Wolf, Tobias Heed, Anna Ball, Holger Cramer, Brigitte Röder, Onur Güntürkün

**Affiliations:** ^1^Department of Biopsychology, Institute of Cognitive Neuroscience, Ruhr-University BochumBochum, Germany; ^2^Biological Psychology and Neuropsychology, University of HamburgHamburg, Germany; ^3^Department of Internal and Integrative Medicine, Kliniken Essen-Mitte, Faculty of Medicine, University of Duisburg-EssenEssen, Germany

**Keywords:** steroid hormones, menstrual cycle, spatial processing, visuotactile integration, crossmodal congruency task

## Abstract

Evidence suggests that spatial processing changes across time in naturally cycling women, which is likely due to neuromodulatory effects of steroid hormones. Yet, it is unknown whether crossmodal spatial processes depend on steroid hormones as well. In the present experiment, the crossmodal congruency task was used to assess visuo-tactile interactions in naturally cycling women, women using hormonal contraceptives and men. Participants adopted either a crossed or uncrossed hands posture. It was tested whether a postural effect of hand crossing on multisensory interactions in the crossmodal congruency task is modulated by women's cycle phase. We found that visuotactile interactions changed according to cycle phase. Naturally cycling women showed a significant difference between the menstrual and the luteal phase for crossed, but not for uncrossed hands postures. The two control groups showed no test sessions effects. Regression analysis revealed a positive relation between estradiol levels and the size of crossmodal congruency effects (CCE), indicating that estradiol seems to have a neuromodulatory effect on posture processing.

## Introduction

Information processing within neural systems is modulated by a diverse array of endo- and exogenous chemical substances (Katz, 1999; Doya, 2002; Marder and Thirumalai, 2002). Steroids and their metabolites, which alter neuronal signaling e.g., by binding to membrane-bound receptors, are among the most powerful neuromodulators (Paul and Purdy, 1992; Melcangi and Panzica, 2006; Melcangi et al., 2011) and have been found to modulate various neurotransmitter systems within the central nervous system, e.g., the GABAergic (Smith et al., 1987a; Smith, 1989; Akk et al., 2005; Hosie et al., 2006), the glutamatergic (Smith et al., 1987b, 1988; Guerra-Araiza et al., 2008), the dopaminergic (Di Paolo, 1994), the (nor-) epinephrinergic (Mahata and Mahata, 1992), and the serotonergic systems (Mahata and Mahata, 1992; but see Hyyppa and Cardinali, 1973). The activity of these molecules, which cross the blood-brain barrier after being synthesized in the steroidogenic glands or are produced by the central nervous system de novo, highly depends on brain region and neuron type (Lambert et al., 2003). Because concentrations of progesterone and estradiol fluctuate dramatically in naturally cycling women within short time intervals (Farage et al., 2008), their effects on information processing have been investigated during different phases of the menstrual cycle (e.g., Hampson, 1990; Bibawi et al., 1995; Rode et al., 1995; Sanders and Wenmoth, 1998; Hausmann et al., 2000; Maki et al., 2002). Especially spatial processing has been found to be modulated by steroid hormones. For example, naturally cycling women have been found to perform significantly better in the mental rotation task during the low estradiol menstrual phase than in the luteal phase (Hausmann et al., 2000).

Traditionally, research on hormonal effects on cognition has focused primarily on the effects of steroid hormones on unisensory (Parlee, 1983; Farage et al., 2008), but not on multisensory processing. Since the merging of senses is a prevalent phenomenon in the human brain (e.g., Hoefer et al., 2013), it is particularly interesting to conduct research on the effects of steroid hormones on multisensory information processing. For example, this would help to understand why sex differences have been observed for spatial remapping processes during multisensory integration (Cadieux et al., 2010) and in general help to explain why large individual differences can be observed in many multisensory integration paradigms.

**Figure 1:**
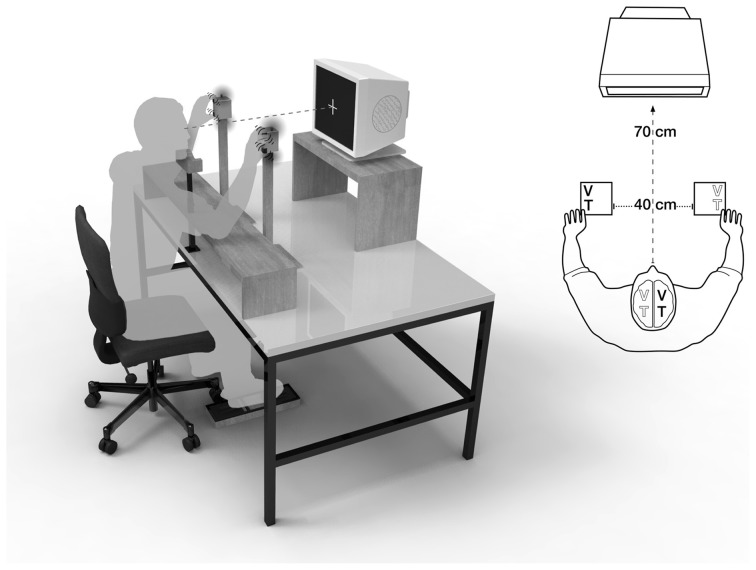
**Visuotactile crossmodal congruency task with uncrossed hands.** A central fixation cross was displayed at a distance of 70 cm, the distance between hands was 40 cm. V, visual distractor; T, tactile target.

**Figure 2:**
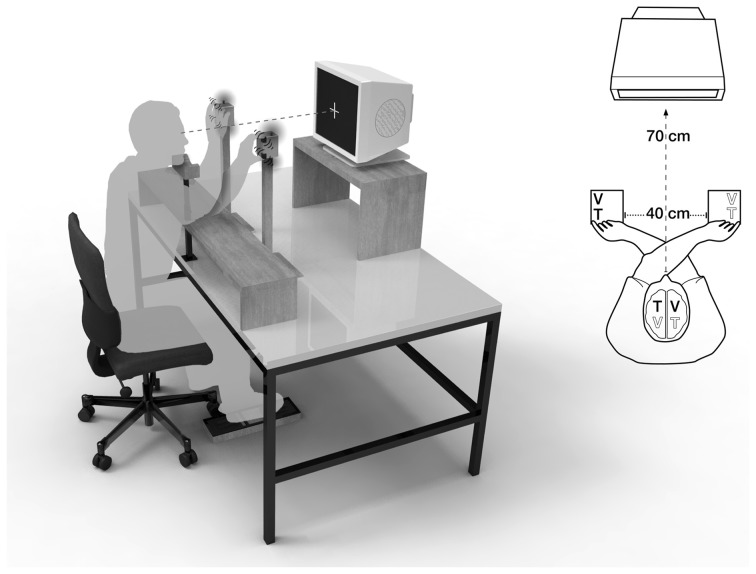
**Visuotactile crossmodal congruency task with crossed hands.** A central fixation cross was displayed at a distance of 70 cm, the distance between hands was 40 cm. V, visual distractor; T, tactile target.

Multisensory interactions are often investigated in tasks that require participants to respond to a target stimulus that is presented in one sensory modality, while (nearly) simultaneously being stimulated by a sensory cue in another modality (e.g., Spence et al., 2004; Schicke et al., 2009; Heed et al., 2010; Bruns et al., 2011a,b). The impact of visual distractors on tactile judgments, for instance, has often been investigated with the crossmodal congruency task, introduced by Spence et al. (1998). In this task, tactile stimuli are presented either at the index finger (“above”) or thumb (“below”) of the left or right hand (see Figures 1 and 2). Tactile targets are accompanied by visual distractors, which are presented at the same time, but at independent locations. Elevation judgments for tactile stimuli are faster and more accurate when the elevation of the visual stimulus is congruent (tactile target and visual distractor both either “above” or “below”), compared to when it is incongruent with the tactile stimulus (tactile target “above” and visual distractor “below” or vice versa), presumably because visual and tactile localization interact. The performance difference between incongruent and congruent visuo-tactile conditions is therefore termed the “crossmodal congruency effect” (CCE).

To investigate crossmodal interactions across hemispheres, the hands are held either uncrossed or crossed. In the crossed posture, the relationship between tactile and visual stimuli is reversed compared to the uncrossed condition. In uncrossed trials, a visual stimulus presented at the same hand as the tactile stimulus presumably maps onto the same hemisphere, whereas a visual stimulus presented at the other hand maps onto the other hemisphere. The opposite is true for crossed trials, because in this posture hands are located within the contralateral visual half-field. Now, tactile and visual stimuli presented at the same hand map onto different hemispheres, and stimuli presented at different hands map onto the same hemisphere (Spence et al., 2001a,b, 2004). Importantly, the CCE is modulated by proximity in external space. Visuotactile interactions are more pronounced for stimuli that are presented in close spatial proximity than for stimuli that are presented further apart from each other (Maravita et al., 2003; Spence et al., 2004). Thus, the CCE is larger when the tactile and the visual stimuli are presented in the same visual hemifield, irrespectively of hand posture. For crossed postures, this indicates that information is relayed between the hemispheres to achieve multisensory integration in an external spatial reference frame. The idea of a predominance of external spatial integration (requiring interhemispheric transfer) over intrahemispheric integration is supported by the findings from a study in which the CCE was determined entirely by proximity of tactile and visual stimuli in external space (Spence et al., 2004). While other studies in healthy participants reported more similar CCE scores for same-side and different-side stimulus pairs in the crossed posture (Maravita et al., 2002; Spence and Walton, 2005; Wolf et al., 2011), Spence et al. (2001a,b) demonstrated in a split-brain patient that an intact corpus callosum is necessary for spatial remapping across hemispheres. Moreover, a recent developmental study showed that children younger than five and a half years did not display a crossed hand effect in the temporal order judgment task, possibly indicating that maturation of the corpus callosum is a prerequisite for spatial remapping (Pagel et al., 2009).

Interestingly, there are studies that indicate that interhemispheric integration processes over the corpus callosum may be one of the subprocesses of spatial remapping that are modulated by sex hormones. According to the hypothesis of progesterone-modulated interhemispheric decoupling, high levels of progesterone attenuate interhemispheric inhibition by decreasing glutamatergic callosal synaptic efficiency (Hausmann and Güntürkün, 2000). Besides interhemispheric inhibition, steroid hormones may modulate interhemispheric integration, i.e., the division of information processing between hemispheres. Bayer et al. (2008) investigated cycle-dependent variations in interhemispheric integration using the Banich-Belger Task (Banich and Belger, 1990). In this task, participants are required to match letters which are presented within or across visual half-fields, according to their physical (e.g., A, A) and semantic (e.g., A, a) identity. Typically, an across-field advantage is observed for the more difficult semantic identity trials, but not for the less demanding physical identity trials. Importantly, Bayer et al. (2008) found that the across-field advantage changed dynamically according to fluctuating levels of steroids in naturally cycling women with a lower across-field advantage being found in the menstrual as compared to the luteal cycle phase. Moreover, a recent visually evoked EEG potential study reported that interhemispheric transfer time, estimated from interhemispheric latency differences of the N170, was longer during the luteal phase as compared to the menstrual phase (Hausmann et al., 2013). In contrast to interhemispheric interaction, there are no studies directly investigating menstrual cycle effects on spatial remapping during multisensory integration yet. Some preliminary evidence, however, comes from a recent study that reported that females show larger tactile temporal order judgment deficits with crossed hands than males, possibly indicating an effect of sex hormones on remapping processes (Cadieux et al., 2010).

Taken together, because fluctuating levels of steroid hormones modulate both spatial processing and interhemispheric crosstalk and visuotactile processing requires callosal connectivity for spatial integration stimuli of conflicting visual and tactile information, performance in the crossmodal congruency task should be affected by the menstrual cycle. Thus, the aim of the present study was to assess whether visuotactile integration is affected by fluctuating levels of steroid hormones. We tested naturally cycling women in the crossmodal congruency task during two cycle phases that are characterized by distinct levels of progesterone and estradiol: the menstrual phase (low levels of progesterone and estradiol) and the luteal phase (high levels of progesterone and estradiol). Additionally, we tested women using hormonal contraceptives and men, for whom, compared to naturally cycling women, relative stable levels of steroid hormones were predicted. Blood samples were taken after every test session to acquire participants' progesterone and estradiol levels. This experimental set-up allowed for the investigation of hormonal effects on both interhemispheric interaction and spatial remapping processes during multisensory integration. For naturally cycling women, we expected that visuotactile interactions would vary across the menstrual cycle due to varying levels of progesterone and/or estradiol. In contrast, visuotactile interactions should be stable across time for women using hormonal contraceptives as well as for men. Specifically, we had the following two working hypotheses:

1. Naturally cycling women, but not the controls groups, should show a cycle-phase-dependent difference in CCE's between trials in which the tactile and visual stimulus map onto different hemispheres as compared to trials were they map onto the same hemisphere.
2. Naturally cycling women, but not the controls groups, should show a cycle-phase-dependent difference in CCE's between the crossed and the uncrossed hands condition. Specifically, we expected a CCE difference in the crossed hands condition between the luteal and menstrual cycle phases, as this condition requires spatial remapping compared to the uncrossed hands condition.

## Methods

### Participants

We tested three groups of participants: 18 naturally cycling women with a regular menstrual cycle of 26–30 days who had not had any hormonal interventions for at least 6 months, 25 men, and 25 women using hormonal contraceptives, who had been applying the NuvaRing for at least 6 months. In contrast to oral contraceptive pills, the NuvaRing's mechanism of action is thought to be constant across time: evenly spread across each day of use, it delivers 120 μg of etonogestrel (a progestin) and 15 μg of ethinyl estradiol (an estrogen). Whereas steroid hormones such as estradiol and progesterone fluctuate over the menstrual cycle in naturally cycling women, we did not expect any significant hormonal fluctuations in women using hormonal contraceptives and men. Mean age did not differ significantly between the three groups of participants [*F*_(2, 67)_ = 1.9, *p* = 0.16, η^2^ = 0.05; see Table 1 for means, standard errors, and ranges].

**Table 1:** **Age, IQ, and LQ of naturally cycling women, women using hormonal contraceptives, and men**.

	**Naturally cycling women**			**Women using hormonal contraceptives**			**Men**		
	***M***	***SE***	**Range**	***M***	***SE***	**Range**	***M***	***SE***	**Range**
Age	25.17	2.89	20–30	23.68	3.28	20–33	25.60	4.36	19–36
IQ	112.76	14.8	86–145	106.36	7.91	91–118	115.04	14.26	97–143
LQ	90.25	12.8	63–100	82.90	17.10	50–100	89.97	11.65	60–100

*Shown are means (M), standard errors (SE), and ranges*.

Prior to testing, we calculated participants' handedness with the Edinburgh Handedness Inventory (Oldfield, 1971). According to the method of Oldfield, laterality quotients (LQs) ranging between −100 (complete left-handedness) and +100 (complete right-handedness) are calculated, with values from −40 to +40 indicating ambidextrality. Only right-handed individuals with LQs = 50 participated in the present study. Mean LQ did not differ significantly between the three groups of participants [*F*_(2, 67)_ = 2.0; *p* = 0.14; η^2^ = 0.05; see Table 1 for means, standard errors, and ranges].

We determined intelligence quotients (IQ) with the Multiple Choice Intelligence Test (Lehrl, 1977), a German standard test. Only individuals with at least average IQs ≥ 80 participated in the study. Mean IQ was marginally lower in the group of women using hormonal contraceptives than mean IQ in the other two groups [*F*_(2, 67)_ = 3.71; *p* = 0.04; see Table 1 for means, standard errors, and ranges].

Participants were recruited by announcements and paid for participation. Furthermore, they were native German speakers, had normal or corrected visual acuity, and were naïve with respect to the experimental hypotheses. All participants gave written informed consent and were treated in accordance with the declaration of Helsinki. The study had been approved by the ethics committee of the Ruhr-University Bochum, Germany.

### General procedure

All three groups of participants were tested twice. Naturally cycling women were tested during the menstrual phase (cycle days 2–5), when low concentrations of estradiol and progesterone were expected, and during the midluteal phase (cycle days 19–23), when high concentrations of estradiol and progesterone were expected (Farage et al., 2008). The individual length of each woman's cycle was taken into account when planning the appointments for testing. Order of testing was randomized across individuals: For half of the women, the first test session took place on days 2–5. For the other half, the first test session took place on days 19–23. Women using hormonal contraceptives were tested in a similar manner, allocating the day of applying a novel NuvaRing to cycle day one. For males, the interval between test sessions was 15–25 days. To control for possible test order effects, the two sessions were assigned randomly to the menstrual cycle phases of naturally cycling women. Because a natural menstrual cycle is not present in women using hormonal contraceptives and men, we will refer to test session A (TA) and test session B (TB) for these two groups.

Immediately after each session, blood samples were taken from all participants. After spinning down the cellular parts of each blood sample, estradiol and progesterone levels were determined by a solid-phase, competitive chemiluminescent enzyme immunoassay (Siemens Diagnostic GmbH, Munich, Germany) with intra- and interassay coefficients of variation (CVs) for a low point of the standard curve being 3.1–7.9% and 4.1–7.8%, respectively. To minimize possible circadian variability in hormone release, the two test sessions took place at the same time of day for each participant (either at 9 am or at 1 pm).

### Crossmodal congruency test

Visuotactile interactions were examined in the Crossmodal Congruency Task with uncrossed and crossed hands (see Figures 1 and 2). The experimental design was modified from Spence et al. (2004). During the task, participants were seated in a darkened room. They focused on a central fixation cross, displayed on a computer screen at a distance of 70 cm. An adjustable chinrest minimized head movements. An adjustable armrest allowed for a comfortable hand-position at eye-level. The armrest was arranged at a distance of 45 cm from the computer monitor. The distance between hands was 40 cm, both in the uncrossed and crossed hands position. Between index finger and thumb of each hand, participants held foam blocks (6 × 6 × 8 cm), which were equipped with two vibrotactile stimulators (Oticon bone conduction vibrators, BC462-100; arranged below finger pads and driven by a 200-Hz sine wave signal), and two red, light-emitting diodes (Vishay Telefunken LEDs TLHR 4405, luminous intensity I_V_ = 10 mcd; arranged beside the vibrators).

Participants were told to focus on the fixation cross and judge the elevation of vibrotactile stimulation while simultaneously ignoring light flashes. Each trial consisted of three 50-ms bursts of vibrotactile stimulations that were separated by 50-ms empty intervals. Tactile stimulations were accompanied by visual stimulations (50-ms light bursts delivered from an LED), which occurred simultaneously, but at independent locations. Responses had to be given as fast and accurately as possible with two foot pedals (Thomann Lead Foot LFD-1). One foot pedal was located beneath the heel, the other beneath the toes of the right foot. Participants lifted their heel to indicate a target at a “lower” position (at the thumb of either hand), and their toes to indicate a target at an “upper” position (at the index finger of either hand). Thus, elevation discrimination was independent of the side from which stimuli were presented. A trial was terminated if no response had occurred within 1.5 s after stimulation. Otherwise, a trial ended with the response of the participant.

Overall, two training blocks (64 trials each) and eight experimental blocks (32 trials each) were conducted. Both training and experimental blocks started with uncrossed hands. Hand posture was changed after each block. Thus, half of the trials were conducted with uncrossed hands, and the other half with crossed hands.

### Data analysis

First, hormone levels were compared between the menstrual and luteal phases in naturally cycling women and between TA/TB for women using hormonal contraceptives and men. Also, hormone levels in naturally cycling women were compared to those in women using hormonal contraceptives. Then, in order to obtain a single measure of overall performance in the Crossmodal Congruency Task, we calculated inverse efficiencies (IE), which control for speed-accuracy trade-offs (Spence et al., 2001a). To derive IE scores, reaction times are divided by the percentage of correct trials, separately for each experimental condition, effectively punishing high error proneness by increasing reaction time. Similarly to previous studies (Spence et al., 2001a,b; Schicke et al., 2009; Heed et al., 2010), we then calculated CCE as the difference in performance in incongruent trials minus congruent trials, measured in terms of IE. CCE are an indicator for the impact of visual distractors on tactile judgments: a large CCE indicates strong crossmodal modulations of tactile location judgments by visual stimuli, whereas small a CCE indicates that visual stimuli modulate tactile location judgments only weakly. To investigate the effects of hormonal fluctuations on the interaction between vision and touch, CCEs were analyzed in a 2 × 2 × 2 × 3 repeated measures Analysis of Variance (ANOVA) with test session (menstrual phase/TA, luteal phase/TB), hemispheric projection (intrahemispheric, interhemispheric), and hand posture (uncrossed, crossed) as within-subject factors and group (naturally cycling women, women using hormonal contraceptives, men) as between-subjects factor. All *post-hoc* tests were Bonferroni-corrected. In order to examine the relationship between levels of gonadal steroids and the CCE, we additionally applied multiple linear regression procedures to test whether estradiol and progesterone reliably predicted the CCE scores.

## Results

### Hormone levels

As expected, both estradiol and progesterone levels were significantly lower during the menstrual compared to the luteal phase in naturally cycling women [*t*_(17)_ = −5.58; *p* < 0.001 and *t*_(17)_ = −10.0; *p* < 0.001, respectively; see Table 2 for means and standard errors]. For women using hormonal contraceptives, neither estradiol nor progesterone levels differed significantly between TA and TB [*t*_(24)_ = 2.06; *p* = 0.051 and *t*_(24)_ = 0.92; *p* = 0.37, respectively]. Moreover, estradiol and progesterone levels of naturally cycling women were significantly higher during the luteal phase than those of women using hormonal contraceptives at TB [*t*_(41)_ = 7.05; *p* < 0.001 and *t*_(41)_ = 9.96; *p* < 0.001, respectively]. In contrast, no differences in hormone levels were observed between the groups when the menstrual phase in naturally cycling women was compared to TA in women using hormonal contraceptives (all *p* < 0.57). For men, neither estradiol [*t*_(24)_ = −0.74; *p* = 0.47] nor progesterone levels [*t*_(24)_ = 0.13; *p* = 0.9] differed significantly between TA and TB. As expected, estradiol and progesterone levels of naturally cycling women were significantly higher than those of men when the luteal phase was compared to TB [*t*_(41)_ = 7.87; *p* < 0.001 and *t*_(41)_ = 11.62; *p* < 0.001, respectively]. When the menstrual phase was compared to TA of men, only the estradiol effect reached significance [*t*_(41)_ = 2.82; *p* < 0.01 and *t*_(41)_ = −1.51; *p* = 0.14, respectively].

**Table 2:** **Means and standard errors (in brackets) of estradiol (pg/ml) and progesterone levels (ng/ml) of naturally cycling women, women using hormonal contraceptives, and men during the menstrual/luteal phase and test session A/B (TA/TB), respectively**.

	**Naturally cycling women**		**Women using hormonal contraceptives**		**Men**	
	**Menstrual**	**Luteal**	**TA**	**TB**	**TA**	**TB**
Estradiol (pg/ml)	38.08 (20.22)	100.89 (46.11)	33.78 (24.11)	23.64 (6.79)	25.23 (9.05)	26.52 (9.49)
Progesterone (ng/ml)	0.26 (0.09)	6.44 (2.64)	0.25 (0.10)	0.23 (0.09)	0.32 (0.15)	0.31 (0.13)

### Visuotactile interactions

Table 3 gives an overview about the average percentage of errors made by the three groups in the different conditions, while Table 4 shows the corresponding average reaction times. Overall, naturally cycling women (16.9% ± 2.19) made more errors than women using hormonal contraceptives (11.08% ± 1.86) and men (7.15% ± 1.86). However, they also reacted faster than the other two groups (naturally cycling women: 345 ms ± 23; women using hormonal contraceptives: 364 ms ± 20; men: 367 ms ± 20). To account for this possible speed-accuracy trade-off, all statistical analyses were conducted using IE scores.

**Table 3:** **Average error rates in percent (standard errors are shown in brackets) for congruent (Con) and incongruent (Incon) trials, differentiated by hemispheric projection (Intra: intrahemispheric; Inter: interhemispheric) and hand posture (UC, uncrossed; C, crossed)**.

			**Naturally cycling women**		**Women using hormonal contraceptives**		**Men**	
			***M***	***L***	**TA**	**TB**	**TA**	**TB**
Intra	UC	Con	13.11	13.37	10.00	8.00	3.81	7.06
			(3.13)	(3.35)	(1.92)	(1.51)	(0.96)	(1.30)
		Incon	18.32	19.01	13.25	10.56	6.75	11.69
			(3.80)	(4.35)	(1.70)	(1.52)	(0.98)	(2.02)
	C	Con	17.71	17.62	11.31	9.88	6.25	8.38
			(3.62)	(3.74)	(2.22)	(1.70)	(1.00)	(1.41)
		Incon	17.62	16.93	11.63	12.06	6.69	7.25
			(3.57)	(4.15)	(1.70)	(1.82)	(0.83)	(1.33)
Inter	UC	Con	14.67	11.63	10.81	9.88	5.25	7.19
			(3.13)	(3.19)	(2.15)	(1.46)	(1.02)	(1.50)
		Incon	16.75	15.54	12.81	10.38	6.25	8.44
			(4.23)	(3.81)	(1.75)	(1.69)	(0.92)	(1.70)
	C	Con	16.84	17.62	12.81	11.19	6.56	8.63
			(3.61)	(3.87)	(2.05)	(1.94)	(0.94)	(1.52)
		Incon	18.58	16.84	11.88	10.88	5.88	8.25
			(3.60)	(3.89)	(2.39)	(2.08)	(0.87)	(1.48)

*For naturally cycling women, the menstrual (M) and luteal (L) cycle phases are shown, while for women using hormonal contraceptives and men test sessions A (TA) and B (TB) are shown*.

**Table 4:** **Average reaction times in ms (standard errors are shown in brackets) for congruent (Con) and incongruent (Incon) trials, differentiated by hemispheric projection (Intra: intrahemispheric; Inter: interhemispheric) and hand posture (UC: uncrossed; C: crossed)**.

			**Naturally cycling women**		**Women using hormonal contraceptives**		**Men**	
			***M***	***L***	**TA**	**TB**	**TA**	**TB**
Intra	UC	Con	317	341	343	354	342	355
			(25)	(36)	(17)	(23)	(16)	(22)
		Incon	332	364	373	370	370	388
			(21)	(35)	(19)	(22)	(15)	(27)
	C	Con	331	371	357	365	363	374
			(25)	(36)	(19)	(23)	(17)	(24)
		Incon	338	359	365	373	369	381
			(33)	(38)	(20)	(23)	(18)	(26)
Inter	UC	Con	327	346	350	360	347	364
			(28)	(27)	(18)	(24)	(15)	(25)
		Incon	338	354	359	360	361	373
			(27)	(32)	(19)	(23)	(17)	(25)
	C	Con	331	367	369	379	368	371
			(25)	(36)	(21)	(22)	(16)	(25)
		Incon	340	357	361	380	369	367
			(27)	(38)	(20)	(25)	(16)	(24)

*For naturally cycling women, the menstrual (M) and luteal (L) cycle phases are shown, while for women using hormonal contraceptives and men test sessions A (TA) and B (TB) are shown*.

**Figure 3:**
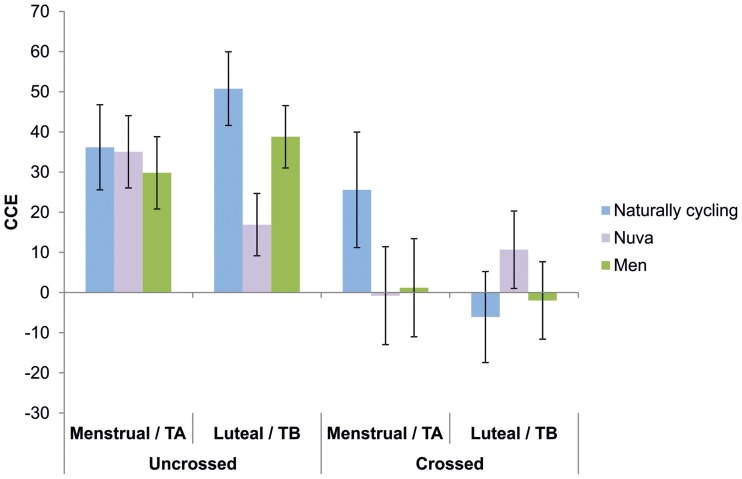
**CCEs for naturally cycling women (blue bars), women using hormonal contraceptives (purple bars), and men (green bars) during the menstrual (M) and luteal phase (L) and different test sessions (TA and TB), respectively.** CCEs are shown for crossed and uncrossed hand posture conditions.

The 2 × 2 × 2 × 3 repeated measures ANOVA revealed a significant main effect of hemispheric projection [*F*_(1, 65)_ = 19.48; *p* < 0.001; η^2^ = 0.23], indicating that the CCE was larger for intrahemispheric (28.93 ± 4.48) than for interhemispheric trials (10.42 ± 3.75). Moreover, a significant main effect of hand posture was observed [*F*_(1, 65)_ = 18.48; *p* < 0.001; η^2^ = 0.22], indicating that the CCE was smaller when participants crossed their hands (4.76 ± 6.05) than when they performed the task with uncrossed hands (34.58 ± 3.68). This hand posture effect was modulated by group and test session/cycle phase as indicated by a significant interaction of test session × hand posture × group [*F*_(2, 65)_ = 4.90; *p* < 0.01; η^2^ = 0.13; see Figure 3]. All other main effects and interactions did not reach significance (all *p* > 0.27).

To further investigate the significant interaction, paired sample *t*-tests comparing menstrual phase/TA to luteal phase/TB were computed for both the crossed hands condition and the uncrossed hands condition in each group. For the crossed hands condition, naturally cycling women showed a significant difference [*t*_(17)_ = 2.25; *p* < 0.05] between menstrual phase (25.56 ± 14,38) and luteal phase (−6.09 ± 11.36), while no difference between cycle phases was observed for the uncrossed hands condition (*p* = 0.42). Also, all comparisons between TA and TB failed to reach significance for both control groups (women using hormonal contraceptives: uncrossed hands: *p* = 0.15; crossed hands: *p* = 0.22; men: uncrossed hands: *p* = 0.35; crossed hands: *p* = 0.71). Thus, an effect of sex hormones on performance in the crossmodal congruency task seems to be limited to the crossed hands condition. To determine the direction of this hormonal modulation on the CCE in naturally cycling women as compared to the controls groups, we analyzed the data from the crossed hands condition using a 2 × 3 repeated measures ANOVA with test session (menstrual phase/TA, luteal phase/TB) and group (naturally cycling women, women using hormonal contraceptives, men) as between-subjects factor. A significant interaction test session × group emerged [*F*_(1, 65)_ = 4.16; *p* < 0.05; η^2^ = 0.11], indicating that in the menstrual phase/TA, the CCE was larger in naturally cycling women (25.56 ± 14.38) than in women using hormonal contraceptives (−0.78 ± 12.20) or men (1.19 ± 12.20). In contrast, in the luteal phase, naturally cycling women (−6.09 ± 11.36) had a reduced CCE compared to the two control groups (women using hormonal contraceptives: 10.78 ± 9.64; men: −1.98 ± 9.64). To test, whether the interaction was driven by the menstrual phase/TA or the luteal phase/TB, we then calculated univariate ANOVAs comparing the three groups for both test sessions. The ANOVA failed to reach significance for both the menstrual phase/TA [*F*_(1, 65)_ = 1.15; *p* = 0.32] and the luteal phase/TB [*F*_(1, 65)_ = 0.74; *p* = 0.48], indicating an overall rather weak effect that seems to be driven by both cycle phases. A similar 2 × 3 repeated measures ANOVA conducted with only the data from the uncrossed condition revealed no significant main effects or interactions (all *p* > 0.15).

Moreover, to exclude the possibility that the interaction in the main analysis was driven by the results of women using hormonal contraceptives, the data in the crossed condition were reanalyzed using a 2 × 2 repeated measures ANOVA with test session (menstrual phase/TA, luteal phase/TB) and group (naturally cycling women, men). The ANOVA revealed a significant main effect of test session [*F*_(1, 41)_ = 5.05; *p* < 0.05; η^2^ = 0.11], as well a trend toward a significant interaction test session × group [*F*_(1, 41)_ = 3.38; *p* = 0.07; η^2^ = 0.07]. *Post-hoc* tests revealed that naturally cycling women had significantly larger CCE's in the menstrual phase (25.56 ± 14.38) than in the luteal phase (−6.09 ± 11.36; *p* < 0.05), while no such effect was observed for men (*p* = 0.71). A similar analysis comparing men and women using hormonal contraceptive revealed no such interaction [*F*_(1, 48)_ = 1.38; *p* = 0.26].

### Relationship between visuotactile interactions and gonadal steroids

Because the interaction between hand posture, session/cycle phase, and group reached significance, we used multiple linear regressions to predict the CCE for crossed and uncrossed conditions from estradiol and progesterone levels for naturally cycling women. For the menstrual phase, a significant linear model of the CCE in the uncrossed different hemisphere condition was revealed [*F*_(2, 17)_ = 5.43; *p* < 0.05; *R*^2^ = 0.34]. Estradiol was a significant predictor (*t* = 2.71, *p* < 0.05), indicating that estradiol levels were positively related to the size of the CCE. Furthermore, in the luteal phase, a trend toward a significant linear model of the CCE in uncrossed trials was observed [*F*_(2, 17)_ = 3.11; *p* = 0.07; *R*^2^ = 0.20]. Again, this model indicated that estradiol levels were positively related to the CCE (*t* = 2.31, *p* < 0.05). No significant relation between progesterone and the CCE was observed. The results of the multiple linear regressions for naturally cycling women are summarized in Table 5. For women using hormonal contraceptives (all *p* > 0.08) and men (all *p* > 0.10), none of the regression models reached significance.

**Table 5:** **Multiple linear regression procedures (standardized β coefficients) for estradiol and progesterone levels as predictors of the CCE in the menstrual and the luteal phase for naturally cycling women**.

**Menstrual**	**Condition**	**Estradiol**	**Progesterone**	***R*^2^**	***p***
Menstrual	Uncrossed	0.54^*^	−0.26	0.42	0.02
	Crossed	−0.13	−0.11	−0.11	0.84
Luteal	Uncrossed	0.53^*^	−0.37	0.20	0.07
	Crossed	0.43	−0.003	0.07	0.22

*Determination coefficients (R^2^) and significance values (p) indicate the goodness-of-fit of the regression model*.

Note:

* *p < 0.05*.

## Discussion

The aim of the present study was to determine the effects of hormonal fluctuations across the menstrual cycle on the interaction between vision and touch. Specifically, it was hypothesized that spatial remapping processes as well as interhemispheric interaction during multisensory integration should be affected by sex hormones. To test these predictions, naturally cycling women were tested in a crossmodal congruency task during the menstrual and luteal phases of the menstrual cycle. Their results were compared with those of women using hormonal contraceptives and those of men. The main finding of our study was that visuotactile interactions changed according to cycle phase in naturally cycling women.

In line with the second hypothesis that naturally cycling women, but not the controls groups, should show a cycle-phase-dependent difference in CCE's between the crossed and the uncrossed hands condition, we found that naturally cycling women showed a significant difference between the menstrual and the luteal phase for the crossed, but not for the uncrossed hands condition. The two control groups showed no test sessions effects. Thus, our results resemble findings from unisensory tasks, for which cycle-dependent variations in sensory perception, e.g., in auditory (Parlee, 1983; Swanson and Dengerink, 1988), olfactory (Asso, 1983; Sommer, 1985; Navarrete-Palacios et al., 2003), visual (Asso, 1983; Parlee, 1983), and tactile acuity (Henkin, 1974; Giamberardino et al., 1997) have been found. Our study is the first to show that such cycle-phase dependent variations also exist for tasks requiring interactions between different sensory modalities. Further analyses revealed that in the menstrual phase/TA the CCE was larger in naturally cycling women than in the two control groups, while the opposite pattern was observed for the luteal phase/TB. Thus, higher levels of steroid hormones seem to lead to a reduction of the CCE when the hands are crossed. Since the CCE represents the performance difference between incongruent and congruent trials, a reduction of this value could implicate that women in the luteal performed better on incongruent trials than those in the menstrual phase, presumably due to more efficient spatial remapping processes.

The results of regression analysis also supported the assumption the CCE is modulated by fluctuating steroid hormones. During both the menstrual and the luteal phases, estradiol levels were positively related to the degree of visuotactile interactions in uncrossed hand trials.

The comparison of those conditions involving a mapping of stimuli onto different hemispheres with those involving a mapping onto the same hemisphere (Spence et al., 2004) allowed for examination of cycle-phase dependent variations in inter- and intrahemispheric visuotactile information processing. However, in contrast to hypothesis 1, the test session × hemispheric projection × group interaction did not reach significance. Thus, naturally cycling women did not show a cycle-phase-dependent difference in CCE's between interhemispheric and intrahemispheric trials when compared to the control groups. Thus, interhemispheric integration in the context of visuotactile processing does not seem to be modulated by steroid hormones to the same extent that is interhemispheric integration in unimodal visual tasks (Hausmann and Güntürkün, 2000; Bayer et al., 2008). However, since the present study is the first work to investigate this phenomenon, more research involving different types of multisensory integration and a wider variety of tasks is needed before any conclusions on this finding can be drawn.

To efficiently perform in the crossmodal congruency task, participants must represent the spatial location of their hand, a process that is mediated by the posterior parietal cortex (PPC) and associated frontal regions (Galati et al., 2010). The PPC is also involved in the transformation of dynamic gaze-centered information into higher-order (e.g., body- or world centered) references frames, a process that is highly relevant for spatial processing (Medendorp et al., 2011). Moreover, it has been found that it is also involved in arm and finger posture processing (e.g., Longo et al., 2010). Thus, it is likely, that the PPC is also relevant for remapping tactile information from an internal to an external reference frame, an assumption that is supported by a number of recent studies in human and monkeys (Lloyd et al., 2003; Graziano and Cooke, 2006; Azañón et al., 2010; Takahashi et al., 2012). Moreover, it has been shown that the PPC is involved in interactions between vision and touch (Bolognini and Maravita, 2007) and it has been proposed that the PPC contains a map of space around the hand (Graziano and Cooke, 2006) that receives input from both visual and somatosensory modalities (e.g., Pasalar et al., 2010; Gentile et al., 2011).

While the present data do not allow definite conclusions about which process is affected by hormonal level differences, they are in line with earlier studies investigating hormonal effects on parietally controlled spatial processes. For example, Hausmann et al. (2000) investigated the performance of naturally cycling women in the mental rotation test, a visuo-spatial task that is known to activate the PPC (Gogos et al., 2010). They found that naturally cycling women performed significantly better in this task during the low estradiol menstrual phase. Moreover, regression analysis revealed a direct negative relation between estradiol level and mental rotation performance, showing that this sex hormone can modulate spatial cognition. This assumption was also supported by an fMRI study measuring brain activity during mental rotation in men and naturally cycling women (Schöning et al., 2007). Here, the authors were able to show that females' parietal and frontal brain activation during mental rotation was significantly correlated with estradiol levels in both the early follicular and midluteal phase of the menstrual cycle.

In contrast to normally cycling women, visuotactile interactions did not depend on test session (TA, TB) in men. In women using hormonal contraceptives, a significant difference between crossed and uncrossed trials was observed in test session A but not test session B. However, the non-significant regression analyses in this group indicate that is unlikely that this result was due to a systematic hormonal effect. Moreover, test sessions were randomly assigned in this group and thus not linked to the day of application of the contraceptive. Nevertheless, these finding still implies that women using the NuvaRing may not be the ideal control group for menstrual cycle studies, since hormonal levels seem to fluctuate to a larger extent than expected. Moreover, a relation between CCE's and estradiol levels was only observed for naturally cycling women, but not for those using the NuvaRing. Thus, our data support the assumption that the behavioral effects of exogenous corticosteroids have specific neural concomitants that are not identical to those of endogenous hormones (Wolkowitz, 1994).

Typically, research focusing on the relation between crossmodal interactions and spatial proximity has revealed that interactions are more pronounced when target and distractor stimuli are presented from the same location than when presented from different locations in space. In general, the effect of hand posture observed in the present study is in line with previous studies (Spence et al., 2004). Evidence suggests that visuotactile space is updated when hands cross the midline, although hands are located in the contralateral visual half-fields (Maravita et al., 2003; Spence et al., 2004). However, because stimuli that are presented at the same hand map onto different hemispheres in the crossed hand posture, processing of such multisensory stimuli involves interhemispheric crosstalk (Spence et al., 2001a,b). Accordingly, other studies have suggested that crossing the hands may lead to intermediate effects (Maravita et al., 2002; Spence and Walton, 2005; Wolf et al., 2011).

In sum, our results revealed that the overall performance pattern of men did not change across time, whereas the interaction between vision and touch depended on cycle phase in naturally cycling women, and may be explained at least partly by the neuromodulatory power of estradiol on spatial processing. Therefore, our findings show that multisensory processing in women is affected by steroid hormones. This result also highlights the importance of controlling for hormonal status when investigating multisensory interaction in women. These findings may also partly explain why females show larger tactile temporal order judgment deficits with crossed hands than males (Cadieux et al., 2010). While Cadieux et al. (2010) did not give information about the hormonal status of their female participants and whether or not they used hormonal contraceptives, it could be speculated that the observed sex difference was mainly driven by female participants in the luteal phase.

### Conflict of interest statement

The authors declare that the research was conducted in the absence of any commercial or financial relationships that could be construed as a potential conflict of interest.
